# Histological study of the effect of different hydration times of bone allograft and xenograft particles on the rate of bone formation in critical size defects in the rat calvarium

**DOI:** 10.1186/s40729-025-00610-0

**Published:** 2025-03-26

**Authors:** Nazila Lashkarizadeh, Mohammad Mohammadi, Zahra Mohyadin, Mahsa Kalantari, Sina Kakooei, Ali Karamoozian

**Affiliations:** 1 Oral and Dental Diseases Research Center, Department of Periodontics, Kerman Dental School, Kerman University of Medical Sciences, Kerman, Iran; 2 Department of Periodontics, School of Dentistry, Rafsanjan University of Medical Sciences, Rafsanjan, Iran; 3 Department of Oral and Maxillofacial Pathology, Oral and Dental Diseases Research Center, Kerman Dental School, Kerman University of Medical Sciences, Kerman, Iran; 4 Department of Biostatistics and Epidemiology, Kerman University of Medical Sciences, Kerman, Iran; 5 Modeling in Health Research Center, Institute for Futures Studies in Health, Kerman University of Medical Sciences, Kerman, Iran

**Keywords:** Allograft, Xenograft, Bone regeneration, Critical size defects, Bone substitutes

## Abstract

**Purpose:**

The aim of this study was to investigate the effect of different bone graft hydration times on bone regeneration.

**Methods:**

Five-mm defects were created on either side of the sagittal plane in the calvaria of 40 rats. In each rat, the right and left defects were filled with allograft (Cenobone^®^) and xenograft (Cerabone^®^) particles, respectively, based on the grouping that was randomly assigned in the study (no hydration of bone graft, 2-minute saline hydration, 10-minute saline hydration, 30-minute saline hydration, and 2-minute blood hydration). Histological and histomorphometrical analyses were performed eight weeks after surgery. The amount of new bone formation, remaining graft, and connective tissue were analyzed using the general linear model (GLM) and Bonferroni test.

**Results:**

There was no significant difference regarding the mean of new bone, remaining graft, and connective tissue between the xenograft samples in different hydration groups. In the allograft groups, the mean new bone formation of the no-hydration and 2-minute saline-hydrated groups was significantly lower than 30-minute saline-hydrated and blood hydrated groups (*P* = 0.03 and *P* = 0.03, respectively). Regarding the variable of the remaining graft particles, the results were almost similar.

**Conclusions:**

The results of this study showed that, the method of bone graft hydration before it is used in treating bone lesions affects osteogenesis. Especially in the case of allograft, rehydration before usage at least for 10 min is recommended.

## Background

Nowadays, different bone reconstruction techniques are employed using bone grafts. An ideal bone graft should be osteogenic, osteoinductive, and osteoconductive [1]. Only autograft has all of these three properties [2, 3]. However, due to restricted access, other types of bone grafts such as allografts and xenografts are used in bone reconstructive surgeries. Most of these grafts only have the osteoconductive property. This property refers to the physical qualities in the graft matrix that provide suitable conditions for the migration of target cells and the formation of new bone.

Allografts are grafts that are made from tissue from the same species [1, 4, 5]. The two most commonly used materials include freeze-dried bone allograft (FDBA) and demineralized freeze-dried bone allograft (DFDBA). FDBA is produced by freezing and dehydration of bone structure without a demineralization process [1, 6]. DFDBA has an additional demineralization stage, and its purpose is to expose the osteoinductive proteins, called BMPs (bone morphogenic protein) [1, 6].

Xenografts are prepared from tissues from animal species [7]. Xenografts are usually only osteoconductive [8]. Bio-Oss^®^ (Geistlich Biomaterials, Wolhusen, Switzerland) and Cerabone^®^ (Botiss Biomaterials GmbH, Zossen, Germany) are two widely used commercial brands of xenografts that are produced through a thermal process [9]. Cerabone^®^ is obtained from bovine cancellous bone. It has a porous mineral structure consisting of hydroxyapatite [9, 10].

It should be kept in mind that the physiochemical properties of bone grafts are effective on their osteoconductive properties [9]. It is desirable that the consistency of the graft used in bone defects should be similar to fresh bone [11]. In the process of preparation of the graft some mechanical properties such as hardness may alter, therefore rehydration of the dry graft is suggested. Rehydration also increases graft plasticity [11].

In a study, non-hydrated and hydrated DFDBA particles were incubated from room temperature to 65 °C for periods of 7 to 35 days. It was found that the osteoinductive potential of DFDBA is influenced by its hydration state. On 7 and 15 days, hydrated DFDBA had more bioactivity than non-hydrated DFDBA. However, after 35 days of incubation, non-hydrated DFDBA outperformed hydrated DFDBA over the entire temperature range [12]. In another study, it was shown that hydration of Bio-Oss^®^ xenograft with saline caused 50% more new bone formation in the first month compared to blood hydration; however, this difference was not observed in the second month [13]. In another experimental animal study, Santos et al. showed that bone graft hydration method may affect bone regeneration and must be done carefully [14].

Considering the dehydration stage in bone graft preparation process (allograft and xenograft), different manufacturers recommend rehydration of bone grafts with normal saline or patient’s blood [14]. However, there is controversy in this field, a detailed protocol has not been provided, and the effect of the hydration time of the graft material on its osteoconductive properties has not been investigated so far. Therefore, in this study, we investigated this issue in critical lesions of rat calvaria.

## Methods

This animal study was designed following the ARRIVE guidelines [15]. The protocol of this study was carried out according to the ethical principles approved by the international committees for the protection of laboratory animal rights and was approved with the ethics code IR.KMU.AEC.1401.012.

Forty healthy adult male rats aged between 92 and 105 days and an approximate weight of 250 to 300 g were obtained from the Laboratory Animal Care and Breeding Center of the Faculty of Medicine, Kerman University of Medical Sciences. The rats were kept in standard laboratory conditions (ambient temperature 22 ± 2 °C, humidity 55 ± 5%, and a 12-hour light-dark cycle) with free access to standard food and water (Nuvilab CR-1, PR, Brazil).

The sample size of 8 in each group was determined by G power software with α = 0.05 and β = 0.80 [13, 16]. So, using block randomization, 40 rats (each with two critical size defects (CSDs), one for allograft and the other for xenograft groups) were initially divided into 5 groups of 8 each. Each group then received one of the interventions (no hydration of bone graft, 2-minute saline hydration, 10-minute saline hydration, 30-minute saline hydration, and 2-minute blood hydration).

**Fig. 1:**
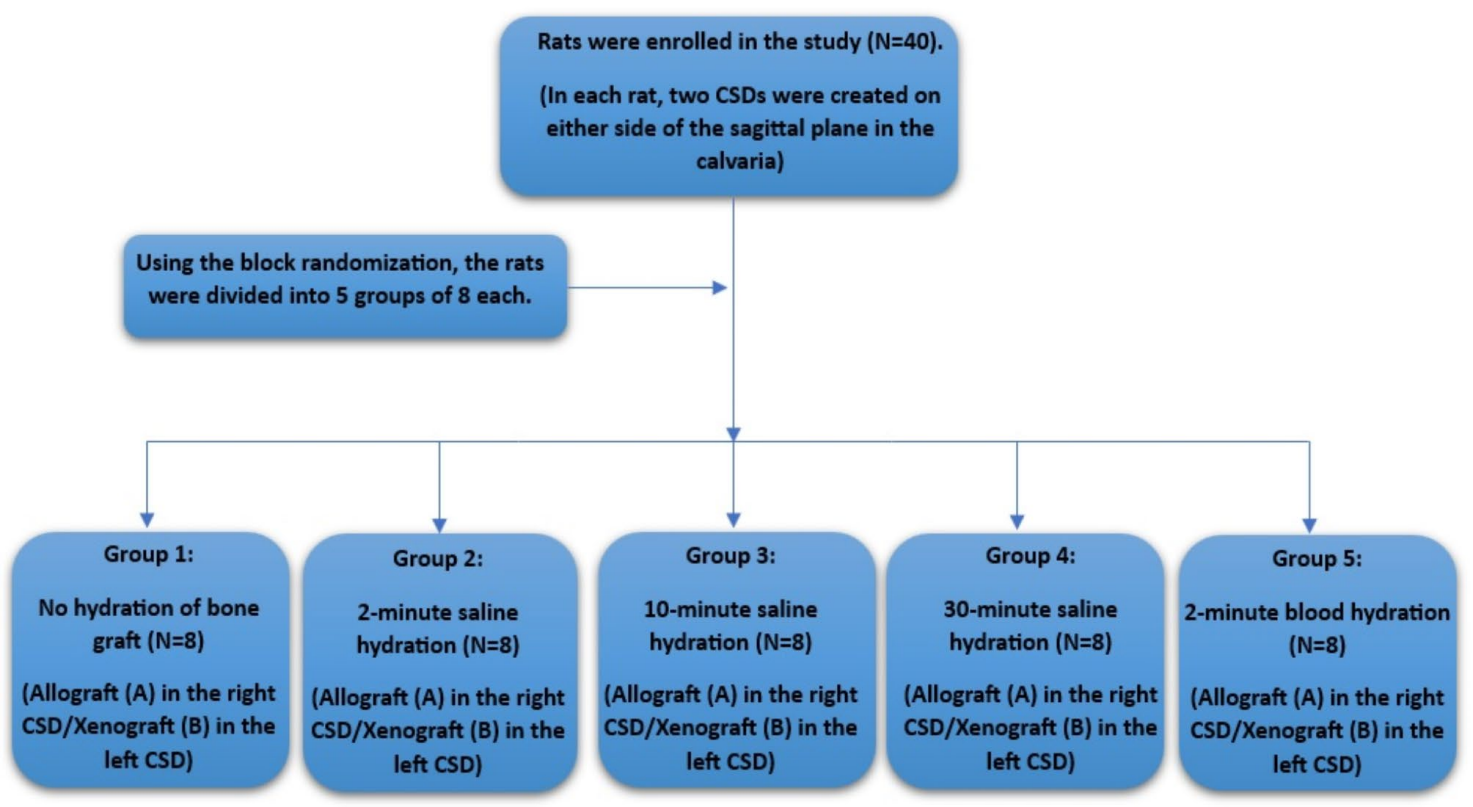
Flow chart of the study groups

Five-mm CSDs were created on either side of the sagittal plane in the calvaria of the rats, and a number was assigned to each rat. In each rat, the right defect was filled with 150–1000-micron corticocancellous FDBA powder (A) (Cenobone^®^, Hamanand Saz Baft Kish, Iran) and the left defect with xenograft particles (B) (Cerabone^®^ granules, Botiss, Germany) with the size of 0.5–1 mm (Fig. 1). All rats were quarantined for 7 days before the start of the experiment. The animals were kept under NPO conditions for 3 h and were prevented from drinking water for 2 h before surgery.

### Surgical procedures

In order to induce anesthesia, ketamine (Ketamine, Alfasan, Woerden-Holland) (65 mg/kg) and xylazine (Xylazin 2%, Alfasan, Woerden-Holland) (7 mg/kg) were used intraperitoneally, and if necessary, anesthesia was continued with 0.25–0.5% isoflurane with 1 L per minute oxygen. The principles of sterility were followed for the surgeon’s hands, the surgical site, and the surgical instruments.

An autoclaved 3-inch stocking was rolled over the rat using aseptic techniques, and the head was pulled out. A No. 40 blade was used to carefully shave the calvarium area and then the area was scrubbed using betadine 7.5% (brown betadine). Then the intended area was disinfected again with 10% betadine solution (green betadine). In order to keep the rat’s head fixed during surgery, it was placed in a rodent stereotaxic device. An incision was made on the mid-sagittal skin using a No. 15 blade. Then, the full-thickness flap was carefully removed by a Molt #9 periosteal elevator (Smart Instru Molt elevator, Pakistan). Two CSDs were created on the calvaria at a distance of 3 mm from the midline on each side, 5 mm in diameter and 2 mm in depth, using a trephine bur (Trephine Bur Dentium, Korea). During the drilling, the surgical area was continuously washed with saline to prevent tissue damage, and great care was taken to avoid damage to the durameter and the fibrous connections attached to the inner surfaces of the cranial bone. the wound was washed with saline and the bone particles were removed by suction [17, 18]. FDBA was used in right cavity in each rat (groups A1 to A5) and xenograft bone was used in left cavity (groups B1 to B5). In each group, the cavities were filled based on the allograft or xenograft hydration method as follows:

1. No hydration– Control group.
2. Two minutes of hydration with 0.9% sterile saline (Darupakhsh, Iran).
3. Ten minutes of hydration with 0.9% sterile saline (Darupakhsh, Iran).
4. Thirty minutes of hydration with 0.9% sterile saline (Darupakhsh, Iran).
5. Two minutes of hydration with blood (blood was obtained from the bone defect site using an insulin syringe).

Then approximately 0.1 cc graft material to fill up to the outer surface of the bone edge was placed in the cavities slowly and without pressure so that the particles did not enter the meningeal space.

After placing the materials into the defects in different groups, the periosteum was returned and sutured in place using a simple single pattern using a 4 − 0 polyglycolic acid suture (Supa, Iran). Then, the skin was sutured using a 3 − 0 nylon suture (Supa, Iran) with a simple single pattern. The surgical area was disinfected with betadine, and tetracycline antibiotic ointment and bandaged. The animal was taken to a warm place to recover from anesthesia. Then, they were transferred to their cages and given food and water. In order to prevent possible infections, 5 mg/kg enrofloxacin 10% (Enrofan, Erfan darou, Iran) and 1 mg/kg keptoprofen 10% (Keptofen, Razak Labratories, Iran) were administered intramuscularly twice a day for first week after surgery and once a day for the second week in all groups. The animals also had free access to standard water and food (Nuvilab CR-1, PR, Brazil). The skin sutures were pulled ten days after the operation.

### Histological analyses

8 weeks [10] after surgery block sections including the area of the original surgical defects were removed and a code based on the number of the rat and type of bone graft was assigned to each sample. The samples were fixed in 10% formalin solution for 3 to 5 days. The decalcification process was done using 10% nitric acid for about 1 to 2 weeks. The acid was changed every day, and this process continued until the bone softened. After the necessary decalcification, the sample was cut with a scalpel and prepared using a tissue processor machine. This preparation takes 16 h and consists of 3 stages of dehydration, clearing with xylenol, and embedding in molten paraffin. Molding was done by an L-shaped aluminum mold with melted paraffin. Then a cut was made using a microtome device with a thickness of 3 to 5 mm in a tissue bath at 55 °C. After deparaffinization, staining with hematoxylin and eosin was done.

The prepared slides were coded, and evaluation was done by a pathologist who was blind to the studied groups.

The histology of the samples was examined using an Olympus YX-100 optical microscope (made in Japan). In this examination, all samples were examined in terms of new bone formation, remains of graft particles, evidence of acute and chronic inflammation, and granulomatous inflammation in response to the foreign object.

### Histomorphometric examination

In order to perform histomorphometric evaluation, micrographs were prepared from each slide under a Nikon-50 optical microscope (made in Japan) connected to a camera. For each sample, one micrograph at 40x magnification was prepared, and histomorphometric analysis was performed on this micrograph. Some micrographs were also prepared at 100x and 400x magnification from various parts of the lesions.

The micrographs were coded according to the slide code and stored in the computer for evaluation using Image J software. The surface of newly formed bone, remains of the graft particles, and soft tissue were calculated. In the next step, the area of each part was divided by the total area and reported as a percentage (for newly formed bone, remains of graft particles, and soft tissue). Histomorphometric evaluation of all samples was done at 40x magnification and all samples were standardized in terms of length, width, and pixels.

In order to differentiate the newly formed bone from the rat’s own native bone, the following criteria were used:

1. Absence of organized mature lamellar bone.
2. Increase in cellularity and new vascularization.
3. Observation of numerous osteocytes.
4. More intense staining by hematoxylin than eosin.

The newly formed bone was differentiated from allograft or xenograft particles based on the presence of cells in living bone lacunae and difference in staining.

### Statistical analysis

The general linear model (GLM) method was used to analyze the studied data according to the objectives of the study. In order to accurately compare the hydration groups according to the structure of the studied response variables, both multivariate and univariate modes were used in this model. Bonferroni correction was also used for pairwise comparison of different groups. The normality of the distribution of the studied data was evaluated using the Shapiro-Wilk test and skewness and kurtosis values. Finally, according to the nature of GLM models, those variables whose distribution was non-normal were also included in this model after the proper conversion. In addition to the GLM model, the generalized estimating equation (GEE) model was also used to compare allograft and xenograft bone grafts. SPSS 22 statistical software was used with 0.05 as the significance level for this study.

## Results

In this study, 40 male rats were studied, none of which were lost during surgical procedures and 8 weeks follow-up period.

### Results of histological analysis

**Fig. 2:**
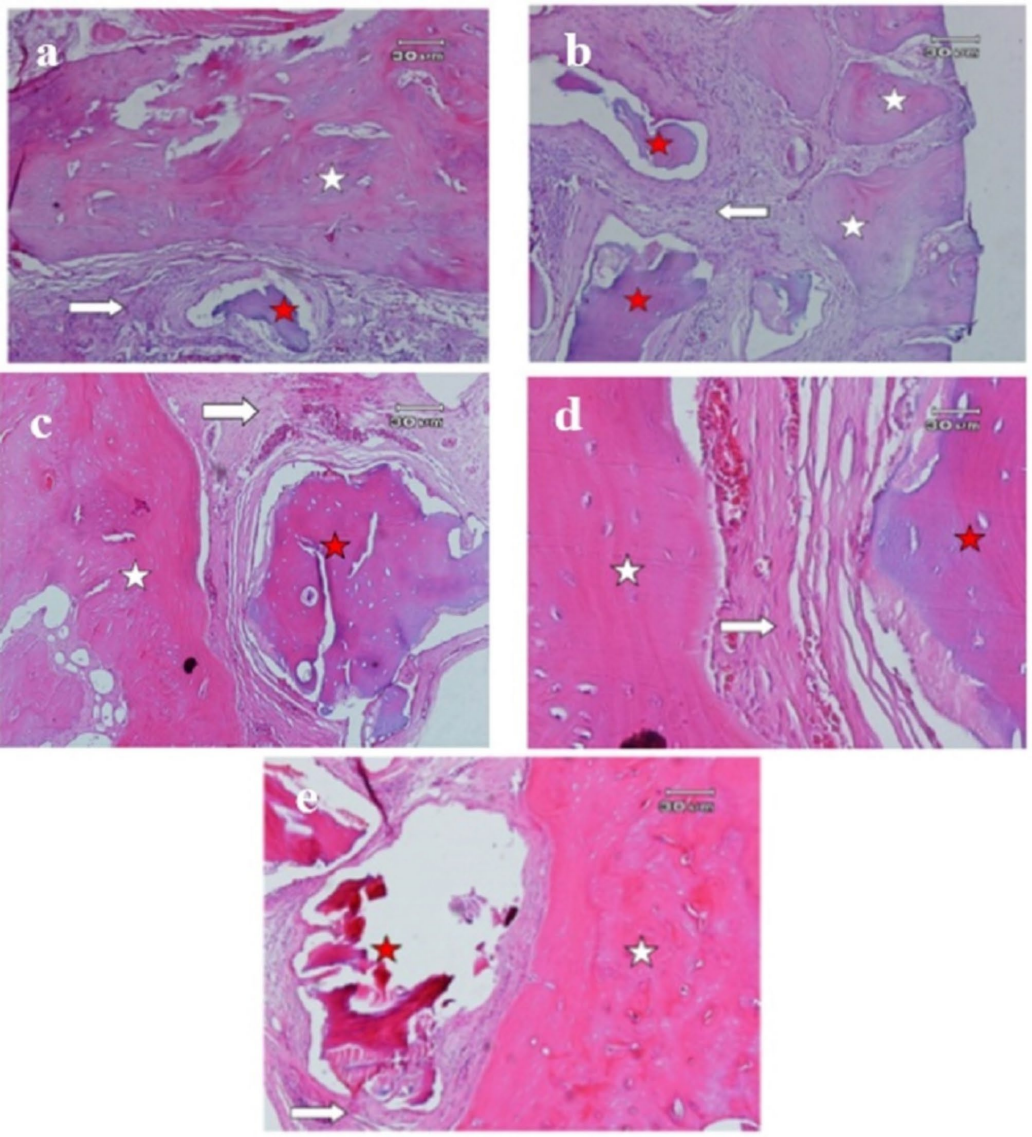
Histological images of critical lesions of calvaria in rats after 8 weeks in different groups of allograft hydration. **a**: No hydration (100x), **b**: 2-minute saline hydration (100x), **c**: 10-minute saline hydration (100x), **d**: 30-minute saline hydration (400x), **e**: Hydration with blood (100x). New bone consisting of numerous lacunae containing osteocytes (white star) is observed in the vicinity of the remains of graft material (red star) and connective tissue (white arrow)

**Fig. 3:**
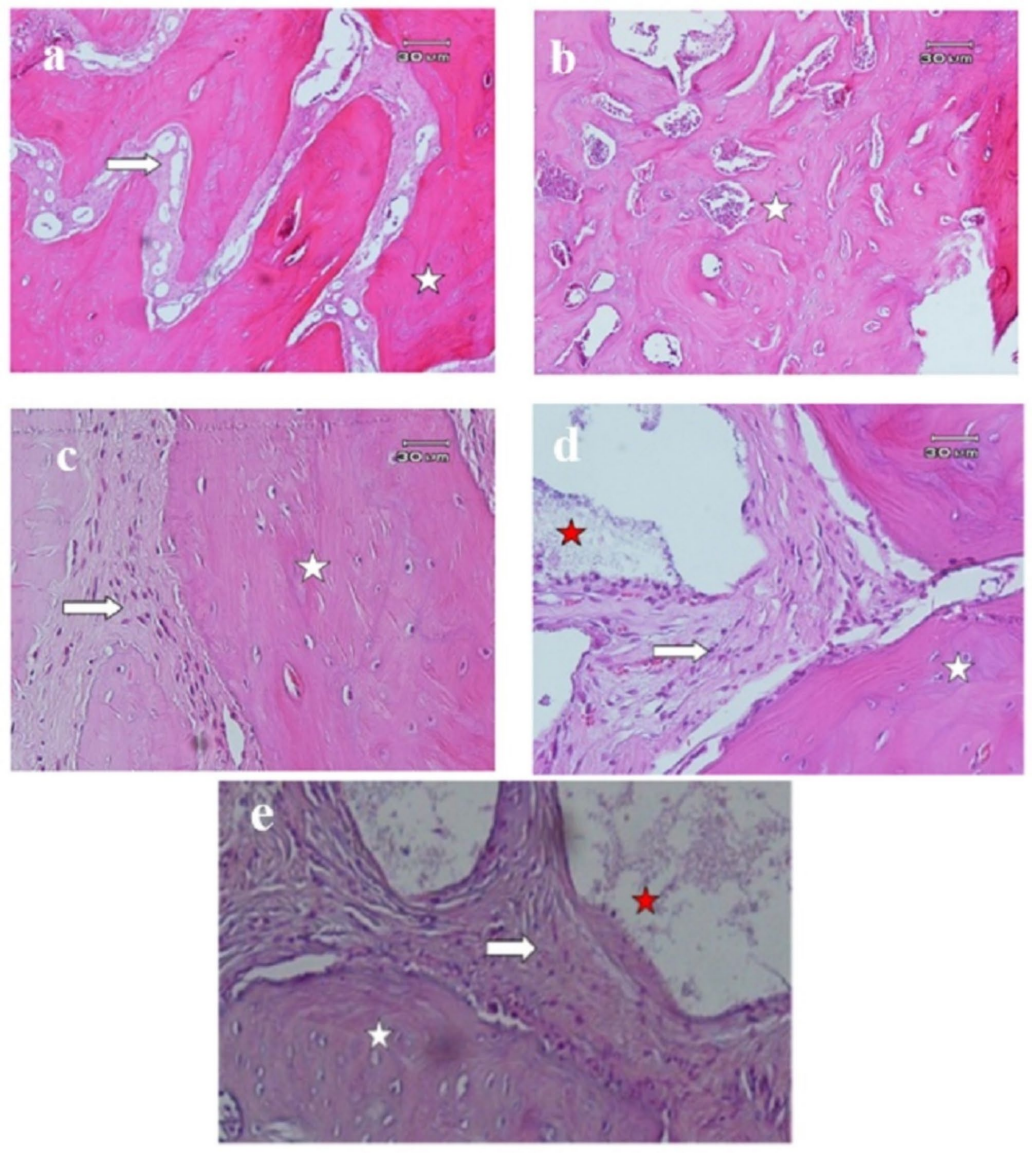
Histological images of critical lesions of calvaria in rats after 8 weeks in different groups of xenograft hydration. **a**: No hydration (100x), **b**: 2-minute saline hydration (100x), **c**: 10-minute saline hydration (400x), **d**: 30-minute saline hydration (400x), **e**: Hydration with blood (400x). New bone consisting of numerous lacunae containing osteocytes (white star) is observed in the vicinity of remains of graft material (red star) and connective tissue (white arrow)

Histological examination of all the samples showed evidence of newly formed bone in the vicinity of allograft and xenograft particles. Allograft and xenograft particles were in close contact with newly formed bone (containing numerous osteocytes). There was no evidence of inflammation and granulomatous reaction in any of the samples (Figs. 2 and 3).

### Results of histomorphometric analysis

In Table 1, the findings related to new bone, remaining graft, and connective tissue have been described separately in xenograft and allograft groups in different hydration conditions as mean, standard deviation, median, and interquartile range. In addition to descriptive findings, the analytical results have also been presented in order to compare different hydration conditions in each of the allograft and xenograft groups separately. There was no significant difference between the mean of new bone, remaining graft, and connective tissue in different hydration groups in xenograft samples. However, there is a statistically significant difference in variables of mean of new bone and remaining graft in different hydration groups in allograft samples (*P* = 0.003 and < 0.001, respectively).

**Table 1:** Results of histomorphometric analysis in critical lesions of rats based on different hydration conditions in the allograft and xenograft groups

		New Bone%		Remaining graft particles%		Connective Tissue%	
		Mean ± SD	Median (IQR)	Mean ± SD	Median (IQR)	Mean ± SD	Median (IQR)
**Xenograft**	Blood hydration	47.16 ± 21.15	41.50 (30.37)	24.94 ± 11.75	22.57 (21.97)	27.90 ± 13.55	23.62 (23.91)
	30 min hydration	40.10 ± 30.67	31.95 (63.36)	25.77 ± 14.48	26.65 (19.78)	34.12 ± 23.32	30.96 (46.43)
	10 min hydration	32.40 ± 28.39	25.18 (45.60)	29.75 ± 14.68	27.53 (21.11)	36.62 ± 13.82	38.44 (25.13)
	2 min hydration	29.77 ± 15.63	32.92 (28.22)	30.19 ± 9.32	31.28 (12.52)	40.04 ± 11.41	42.87 (18.66
	No hydration	25.83 ± 16.86	23.25 (33.01)	26.93 ± 7.92	27.58 (13.27)	48.49 ± 14.87	45.79 (29.33)
***P*** **-value**		0.38		0.87		0.15	
**Allograft**	Blood hydration	45.42 ± 18.33	51.58 (30.20)	21.12 ± 8.60	20.40 (16.77)	34.46 ± 16.82	26.48 (25.83)
	30 min hydration	45.81 ± 24.29	53.07 (50.83)	17.90 ± 12.11	11.86 (19.38)	36.17 ± 15.98	33.77 (28.72)
	10 min hydration	34.69 ± 18.45	30.69 (32.81)	27.12 ± 10.91	27.23 (21.62)	38.18 ± 9.84	39.01 (12.41)
	2 min hydration	17.84 ± 8.79	13.28 (13.00)	35.57 ± 6.11	36.68 (10.49)	44.80 ± 5.98	46.40 (7.68)
	No hydration	17.20 ± 15.78	11.79 (5.67)	40.19 ± 10.98	42.18 (17.45)	42.60 ± 11.15	40.62 (17.17)
***P*** **-value**		0.003*		< 0.001*		0.44	

SD: standard deviation, IQR: interquartile range, * *P* < 0.05

According to the results related to the multivariate model for the allograft group (Table 2), it is clear that there is a statistically significant difference between different hydration groups in at least one of the variables of new bone, remaining graft, and connective tissue. In order to clearly understand in which of these three variables there is a statistically significant difference between the hydration groups, we referred to the univariate test, and the results are presented in Table 1.

**Table 2:** Multivariate GLM

Group	Test	Estimated value	F Statistic	*P*-value
**Xenograft**	**Pillai’s trace**	0.34	1.10	0.37
	**Wilks’ lambda**	0.69	1.11	0.36
	**Hotelling’s trace**	0.42	1.12	0.36
**Allograft**	**Pillai’s trace**	0.57	2.07	0.02
	**Wilks’ lambda**	0.47	2.40	0.01
	**Hotelling’s trace**	1.02	2.70	0.004

GLM: General linear model

According to the results of Table 3, allograft hydration groups have been compared against each other in pairs to determine the groups that are significantly different. There was a significant difference between the new bone average of the no-hydration group and the 30-minute saline hydration and blood hydration groups (*P* = 0.03 and *P* = 0.03, respectively). In addition, there was a statistically significant difference between the average percentage of new bone in the 2-minute saline hydration group and the 30-minute saline hydration and blood hydration groups (*P* = 0.03 and *P* = 0.04, respectively). Regarding the variable of amount of remaining graft material, the results were the same.

**Table 3:** Pairwise comparison of different hydration groups for the variables of new bone and remaining graft particles in allograft samples

Pairwise comparison	New bone	Remaining graft particles
	*P*-value	
**No hydration– 2-min hydration**	0.62	0.53
**No hydration– 10-min hydration**	0.58	0.07
**No hydration– Blood hydration**	0.03*	0.001*
**No hydration– 30-min hydration**	0.03*	0.003*
**2-min hydration– 10-min hydration**	0.07	0.24
**2-min hydration– Blood hydration**	0.03*	0.01*
**2-min hydration– 30-min hydration**	0.04*	0.04*
**10-min hydration– Blood hydration**	0.56	0.22
**10-min hydration– 30-min hydration**	0.47	0.12
**Blood hydration– 30-min hydration**	0.88	0.75

* *P* < 0.05

According to the results of Table 4, when the difference between allograft and xenograft was evaluated in different sub-groups (hydration status), it was found that there was a significant difference between the two groups only in the amount of remaining graft in no-hydration conditions, and in other cases, there was no statistically significant difference at 95% confidence level.

**Table 4:** Comparison of variables of amount of new bone, remaining graft particles, and connective tissue between allograft and xenograft samples based on different hydration groups

	New Bone%			Remaining graft particles%			Connective Tissue%		
	Allograft	Xenograft	*P*-value	Allograft	Xenograft	*P*-value	Allograft	Xenograft	*P*-value
	Median (IQR)								
**Blood hydration**	51.58 (30.20)	41.50 (30.37)	0.86	20.40 (16.77)	22.57 (21.97)	0.36	26.48 (25.83)	23.62 (23.91)	0.40
**30-min hydration**	53.07 (50.83)	31.95 (63.36)	0.69	11.86 (19.38)	26.65 (19.78)	0.26	33.77 (28.72)	30.96 (46.43)	0.84
**10-min hydration**	30.69 (32.81)	25.18 (45.60)	0.85	27.23 (21.62)	27.53 (21.11)	0.69	39.01 (12.41)	38.44 (25.13)	0.80
**2-min hydration**	13.28 (13.00)	32.92 (28.22)	0.08	36.68 (10.49)	31.28 (12.52)	0.19	46.40 (7.68)	42.87 (18.66	0.31
**No hydration**	11.79 (5.67)	23.25 (33.01)	0.30	42.18 (17.45)	27.58 (13.27)	0.02*	40.62 (17.17)	45.79 (29.33)	0.38

IQR: interquartile range, * *P* < 0.05

## Discussion

This study was conducted with the aim of histologically investigating the effects of different hydration times of bone allograft and xenograft particles on the amount of bone formation in defects with the critical size of 5 mm in rat calvaria within 8 weeks. This time period is suitable to check the rate of new bone formation, bone maturation, and amount of bone material absorption so that clinically significant differences can be analyzed [10].

The results of this study showed that, in 8 weeks, there was no significant difference in new bone formation in each hydration group between FDBA and xenograft.

However, the amount of new bone formation between variant hydration groups was different and within a wide range in each group. In the study of Susin, in which different types of bone grafts in 8-mm critical lesions of rats, were histologically evaluated, similar to the present study a wide range of percentages of new bone formation were reported in each group, and no statistical difference was observed between grafts in terms of the rate of new bone formation, remaining graft, and connective tissue. Considering that in this study, the evaluation was done within 4 weeks, the rate of new bone formation was lower in all groups than in the present study [10]. The study of Borie et al. investigated FDBA in 8-mm critical lesions of rabbit calvaria. The formation rate of new mineralized tissue in 45 and 60 days was 44% and 69%, respectively, and as time passed, it increased up to 90 days. These statistics are consistent with the average amount of new bone formation in the 30-minute saline hydration group and blood hydration group in our study [19]. In the study of Mellonig et al. on critical lesions of guinea pigs, the average rate of new bone formation using FDBA after 42 days was 40.34% [20].

The results of the present study showed that in both allograft and xenograft cases, with the increase in the hydration time of the graft particles with saline and in the blood group, the amount of new bone formation in CSDs increased, while the amount of remaining graft particles and connective tissue decreased. However, this difference was significant only for allograft cases in the variables of new bone formation and remaining graft particles between particles hydrated with blood and those hydrated with saline for 30 min with the no-hydration and 2-minute hydration groups. Regarding xenograft, no significant difference was observed between the mean of new bone, remaining graft, and connective tissue in different hydration groups. Considering that xenograft has a three-dimensional porous network structure that quickly absorbs water, blood, and serum, and is rapidly hydrated, this observation seems reasonable [21, 22]. A laboratory study showed that xenograft is as hydrophilic while it may be slightly different in terms of properties such as having nano-pores or in physical, chemical, and crystalline structure [9]. In the case of FDBA, due to the fact that it is dehydrated during its production using a freeze-drying process, adequate hydration of this biomaterial is important before use in lesions.

In the study of Santos et al., it was observed that biphasic ceramic bone graft (SBC) hydration with saline compared to hydration with blood significantly increased the volume percentage of new bone (63.65 ± 8.01 compared to 52.51 ± 13.42) and decreased the volume percentage of connective tissue (27.81 ± 9.08 compared to 40.31 ± 16.81) in the extracted tooth socket of rats after 42 days [14]. The effect of hydration time that was not mentioned in this study, type of defects and graft material can be mentioned among the reasons for the difference of results between this study and the present study.

In the study done by Durual et al. (2021), the amount of new bone formation was evaluated after the application of deproteinized bovine bone mineral particles embedded in collagen (DBBM-C) xenograft that was hydrated with 0.9% saline or blood (taken from the ear vein) for 30 to 120 s. In a laboratory examination, prepared DBBM-C blocks were fully hydrated in saline in 30 s while 120 s of hydration with blood failed to hydrate the central area of the blocks. In addition, in terms of bone graft handling, blood hydration led to a gradual decrease in stiffness by 2.5 times, and saline hydration led to up to six times decrease in stiffness within 30 s. In the histomorphometric analysis of xenografts in critical lesions of rabbit calvaria, saline hydration resulted in 50% more bone regeneration compared to blood hydration, in the first month. However, at later times, this difference was not observed, and the morphology and maturity of the new bone were the same in both conditions [13]. Due to the fact that the analysis of the present study was conducted over a period of 2 months, it is in line with this study.

The results of the present study showed that in the no-hydration group, the percentage of remaining graft in the allograft group (42.18 ± 17.45) was significantly higher than the xenograft group (27.58 ± 13.27). The lack of hydration of the allograft may influence physical properties including consistency, which may have affected the migration, adhesion, and differentiation of osteoclast cells; therefore, instead of ideal process of graft consolidation which includes dissolution and replacement of graft particles with new bone, connective tissue was formed near the graft particles.

The process of bone formation in GBR depends on various factors, such as the provision of suitable environmental conditions for the migration of precursor cells, differentiation of osteoclasts, differentiation and attachment of osteoblasts. The differentiation of osteoclasts and their activity led to the decomposition of the bone graft, and osteoblasts cause the deposition of new bone in the vicinity of the graft [23]. An ideal bone graft should have high hydration ability to facilitate the movement and growth of angiogenic factors by providing an isosmotic environment. It should have porosity, chemical composition, and mechanical properties similar to the native bone to allow infiltration, adhesion and growth of osteogenic cells. With hydration, these properties of the graft are improved, and a more favorable environment suitable for new bone formation is provided [24]. It has been reported that in compression tests, dry allograft has maximum effective stress and maximum deformation, significantly higher than fresh bone. These values become similar to fresh bone after hydration [11].

In the present study, in both xenograft and allograft cases, in the groups with graft particles hydrated with blood, the histopathological results were favorable, and the average percentage of new bone formation was 47.16 ± 21.15 and 45.42 ± 18.33, respectively. In the bone regeneration process, clot formation and preservation are the key to success. Clot can be formed to act as an anchor for growth factors and cell recruitment. Clinically, it is generally recommended to immerse the scaffolds in blood from the surgical site to promote these mechanisms [13].

In this study, defects with a critical size of 5 mm in calvaria of rats were used as a model [13, 18, 25–27]. This model is a suitable preclinical protocol to evaluate the efficiency of biomaterials before human applications [28]. However, the defect size has an influence on the results of this study (5 mm is more favorable for regeneration than other studies using an 8 mm defect). So, this model cannot completely and flawlessly simulate the clinical characteristics of human patients due to the biomechanical and geometrical differences. Therefore, the results obtained from this research should be generalized to clinical conditions and in humans with full caution, as they are more comparable to self-containing defects that work reliably with particulate material. Not examining normal saline hydration times between 10 and 30 min and not examining other grafts can be considered other limitations of this study.

According to the present study, complete hydration of the graft is necessary and may be effective on cell differentiation and connection and osteogenesis in the GBR process. Hydration of graft particles especially in allograft with saline for at least 10 min, preferably 30 min, or with blood is suggested before use in lesions to increase new bone formation and improve bone quality. Improving the quality of bone increases the contact surface with the implant and creates a bone that has the ability to withstand forces [29].

## Conclusion

The results of this study showed that biomaterial hydration method, with saline or blood, and different hydration times may affect osteogenesis in periodontal defects. So, adequate hydration of xenograft and allograft particles is suggested before use in lesions. Hydration time is more critical about allograft compared to xenograft, and hydration with saline for 30 min or with blood increased new bone formation compared to the no-hydration and 2 min saline hydrated groups in allograft. No difference was observed between hydration with blood and 30-minute hydration with saline in allograft and xenograft groups.

## Data Availability

The data sets generated and analyzed during the current study are available from the corresponding author on reasonable request.

## Abbreviations

FDBA: Freeze-dried bone allograft
DFDBA: Demineralized freeze-dried bone allograft
BMP: Bone morphogenic protein
GLM: General linear model
GEE: Generalized estimating equation
DBBM-C: Deproteinized bovine bone mineral particles embedded in collagen
GBR: Guided bone regeneration
